# A Review of the Potential Role of Human Cytomegalovirus (HCMV) Infections in Breast Cancer Carcinogenesis and Abnormal Immunity

**DOI:** 10.3390/cancers11121842

**Published:** 2019-11-22

**Authors:** Jürgen Geisler, Joel Touma, Afsar Rahbar, Cecilia Söderberg-Nauclér, Katja Vetvik

**Affiliations:** 1 Department of Oncology, Akershus University Hospital (AHUS), 1478 Lørenskog, Norway; juergen.geisler@medisin.uio.no (J.G.); Joel.Touma@ahus.no (J.T.); 2 Institute of Clinical Medicine, University of Oslo, Campus Akershus University Hospital (AHUS), 1478 Lørenskog, Norway; 3 Department of Breast and Endocrine Surgery at Akershus University Hospital (AHUS), 1478 Lørenskog, Norway; 4 Department of Medicine, Division of Microbial Pathogenesis, Bioclinicum, Karolinska Institutet, 17176 Stockholm, Sweden; afsar.rahbar@ki.se (A.R.); cecilia.naucler@ki.se (C.S.-N.); 5 Department of Neurosurgery, Karolinska University Hospital, 17176 Stockholm, Sweden

**Keywords:** cancer, breast cancer, triple negative breast cancer, human cytomegalovirus, tumor associated macrophages, T cells, dendritic cells, tumor immunology, check-point inhibitors, antiviral treatment

## Abstract

Previously recognized classical human onco-viruses can regulate complex neoplastic events, and are estimated to play a role during carcinogenesis in 15–20% of cancer cases. Although the DNA and gene products of several viruses have been found in breast tumors, none of the classical onco-viruses have definitely been linked to the initiation of breast cancer. However, recent evidence shows that human cytomegalovirus (HCMV) gene products are found in >90% of tumors and metastases of breast cancers, and their increased expression can be correlated to a more aggressive breast cancer phenotype. Supporting the active role of HCMV in breast cancer, a specific HCMV strain, HCMV-DB, was recently shown to exert oncogenic transformational activity in breast epithelial cells in vitro, and to give rise to fast-growing, triple-negative breast tumors when injected into immune deficient mice. The same observation holds true for clinical studies implying increased HCMV protein expression in triple negative breast cancer biopsies. In addition to functionally being able to hijack tumor-promoting cellular events, HCMV is known to exhibit a wide range of immunosuppressive effects, which can have radical impact on the tumor microenvironment. HCMV infected cells can avoid recognition and elimination by the immune system by orchestrating polarization of immunosuppressive type II macrophages, preventing antigen presentation, by expressing T cell inhibitory molecules, and possibly, by the induction of regulatory T (Treg) cell responses. These actions would be especially deleterious for the antigenic activation and proliferation of tumor specific CD8^+^ cytotoxic T lymphocytes (CTLs), whose effector functions have recently been targeted by successful, experimental immunotherapy protocols. The recognition of alternative causes and drivers of breast cancer is a pivotal research topic for the development of diagnostics and novel, effective preventive and therapeutic strategies targeting both tumor cells and their microenvironments.

## 1. Background

Breast cancer (BC) is the most common cancer in women worldwide. Approximately 15–20% of all breast cancers are of triple-negative BC (TNBC) subtype, which displays negative immuno-histochemical expression patterns of hormone receptors (estrogen receptor (ER), progesterone receptor (PR)), and human epidermal growth factor receptor 2 (HER2). The TNBC subtype is associated with poorer long-term prognosis when compared with other BC types. Once metastatic, TNBC tumors are often resistant to chemotherapy, and patients show a median overall survival of 12–18 months only [1].

Known risk factors of BC are believed to affect the onset and severity of BC, and include factors related to age and life-style as well as positive family history with or without known genetic aberrations. The classical cancer risk factors might create a tumor-favoring cellular environment, where oncogenic viruses may reside and increase their oncogenic potential. Even if historically controversial, rising evidence reports the frequent presence of human cytomegalovirus (HCMV) in BC [2,3], BC derived metastatic brain tumors [3,4], and in breast ductal carcinoma in situ [2], suggesting a potential association between HCMV infection and breast carcinogenesis in certain subgroups of patients [2,3,4]. This review will highlight the potential complex functional role of HCMV infection in human BC, focusing on TNBC, and attempt to summarize the contemporary scientific knowledge on the subject.

## 2. Human Cytomegalovirus (HCMV)

Human cytomegalovirus (HCMV), also known as human herpes Virus 5 (HHV-5), is a ubiquitous, opportunistic DNA virus carried by up to 90% of the adult population worldwide. After primary infection, HCMV establishes a life-long latency predominantly in the CD34^+^ hematopoietic progenitor cell population residing in the bone marrow. It is assumed that HCMV may intermittently reactivate in a stochastic manner unless continuously controlled by the host immune system. For this reason, the virus is considered harmless in healthy individuals, but may cause life-threatening complications in immunocompromised patients. Latent HCMV infection can be reactivated during an inflammatory process when the progenitor cells differentiate into monocyte/infiltrating macrophages or dendritic cells, and these cells can disseminate the virus to peripheral organs [5]. Reactivated HCMV, carried by these inflammatory cells, can reach all body tissues, and infect and replicate in a broad number of cell types [6]. The infection is further transmitted by all body fluids, including saliva and breast milk [7]. Ninety-percent of breast milk samples from HCMV seropositive women contain the virus, and that results in about 30% HCMV prevalence in children at one year of age. Nursing and parental contact, therefore, constitutes an important route to acquiring the HCMV infection in early infancy or childhood [7].

## 3. Oncogenic Properties of HCMV

HCMV encoded proteins display diverse oncogenic functions. Upon entry into the host cell, tegument proteins of the HCMV virion, such as pp65 and pUL48, are released, disabling cellular intrinsic and innate immune responses, and promoting enhanced metabolic activity of the host cells [8]. These HCMV-encoded proteins may enable the cells to surpass the G1-phase to facilitate rapid cell-division [8]. Through upregulation of anti-apoptotic genes and downregulation of pro-apoptotic genes, cells enter a state of enhanced survival.

After the entry of viral DNA into the cell nucleus, cellular RNA polymerases I and II (Pol I and II) are employed to transcribe the viral genes by binding to the major immediate early promoter (MIEP) [9]. The first genes that are expressed are the immediate early (IE) genes, producing the IE72/IE1 and IE86/IE2 proteins. The IE proteins act as transcription factors controlling both early and late viral gene expression, and direct host gene expression. Both proteins are necessary to establish lytic infection and are crucial for viral re-activation from latency [8,10]. Lytic HCMV infection leads to a dysregulated cell cycle, and the IE gene products interfere with key cellular factors, including retinoblastoma protein family (Rb), cyclins, p53, Wnt, phosphatidylinositol 3-kinase/Akt, human telomerase reverse transcriptase (hTERT), and NF-κB to increase the immortal properties of infected cells [11]. These pathways are commonly activated in cancer cells. Activation of mitogenic signals, delivered by proto-oncogenes such as Fos and Myc, can be induced by the IE1 and IE2 proteins in HCMV infected cells [12]. Moreover, the *MYB* gene is induced in HCMV infected cells resembling the enhanced *MYB* gene expression in HPV-related carcinoma [11]. In addition to the mitogenic signals, HCMV infection causes chromosomal aberrations through deterioration of DNA repair pathways, resulting in genetic instability in the infected cells [13,14]. This fuels the development of genetic mutations.

Four HCMV-encoded, G-protein-coupled-receptor (GPCR)-like proteins, US27, US28, UL33, and UL78, display important oncogenic functions [15]. G-proteins activate both metabolic and oncogenic key signaling pathways, such as cAMP and the PI3K signaling pathways, of which the latter is critical for the emergence of anchorage-independent growth and oncogenic transformation of epithelial cells [11,16]. The HCMV-β2.7 early gene transcript, is a long non-coding (lnc) RNA that interacts directly with complex I of the respiratory chain in mitochondria, preventing mitochondria-induced cell death by inhibiting Fas-ligand interactions and granzyme B by binding to caspase 8, improving the oxidative capacity and maintaining energy production in the infected cells [17].

HCMV has also developed several ways to manipulate the innate and adaptive immune responses to decrease its immune surveillance and improve its chances of surviving in its immunocompetent host, which may well account for the important immune evasive mechanisms in the HCMV-infected cancer cells. HCMV encodes multiple proteins that modulate NK cell recognition of the infected cells [18], and increase CD8+ T cell tolerability for the viral proteins. HCMV encoded proteins can stimulate the development of an immature phenotype of dendritic cell (DC), which reduces the activation of CD4+ T-cell responses [19], and additionally, decreases the elimination of infected cells by CD8+ cytotoxic T cells. 

## 4. HCMV in Breast Cancer

Increasing evidence demonstrates that HCMV is present in breast carcinoma in situ. The virus is found in >90% of early breast cancers and in >90% of metastatic deposits from breast cancer [3,4,20,21]. Similar observations have been reported from studies of other cancers, especially those arising from epithelial or neuro-epithelial cells [2,3,22,23]. HCMV-positive cells are typically restricted to cancer cells in primary tumors and metastasis, but viral proteins are sometimes also observed in tumor associated inflammatory cells. In sharp contrast, virus-positive cells are generally not found in adjacent normal tissues in the same patient or in healthy individuals, with one exception [3,4]. HCMV can be found rarely in the breast cells of healthy women [2]; this is expected as the virus is known to be present in the breast and can reactivate and produce infectious virus in 90% of HCMV seropositive breast-feeding women [7]. This could be a potentially evolutionarily conserved way to secure viral transmission. It is important to point out that the occurrence of an active HCMV infection with protein production is very rare in tissues of healthy individuals. Therefore, the presence of an active HCMV infection in breast cancer is intriguing and needs further investigation.

Despite the known onco- and immunomodulatory effects, HCMV has not been recognized as a traditional tumor-initiating virus or a classical onco-virus. Results from past publications investigating the presence of HCMV in breast or other cancers have been inconsistent, which was discussed by us in more detail in another recent review article [24]. These discrepancies can, to some extent, be explained by difficulties to detect viral DNA/RNA by PCR based techniques in clinical tumor samples or by other technical reasons [20,25,26]. The discussion of whether HCMV plays a role in cancer or not, has been further fueled by the lack of direct evidence of oncogenic transformation induced by HCMV, since most of the HCMV strains examined are unable to transform normal human cells in in vitro cultures. However, a recently published study by Kumar et al. provides evidence that HCMV infection of primary human mammary epithelial cells (HMECs) with a clinical strain, named HCMV-DB, can transform primary HMECs in vitro [27]. The transformed cells display a triple negative phenotype with low expression of luminal markers (KRT19, KRT18, GATA3, and TFF1) [11]. The transformed cells expressed an HCMV IncRNA4.9 genomic sequence and gave rise to fast-growing triple negative breast tumors when injected in NOD scid gamma (NSG) mice. An analogous HCMV encoded IncRNA4.9 sequence was detected in tumor biopsies of patients with BC, but not in healthy human breast tissues [27]. In addition, several, recently published clinical studies reported on the significant presence of HCMV gene products in TNBC, but the virus was not found in adjacent normal tissues [23,28].

**Figure 1:**
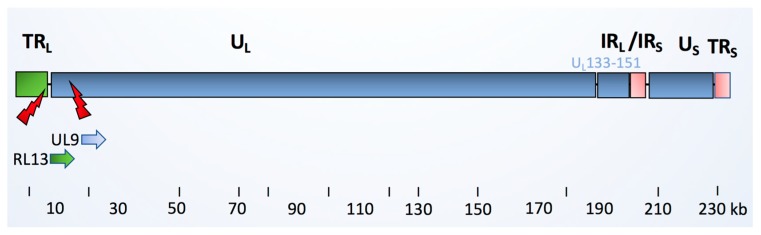
Schematic picture of the HCMV clinical isolate. The HCMV viral genome consists of linear double stranded DNA, ranging from 220 to 240 kB in length. The genome is comprised of unique long (UL) and a unique short (US) regions, which are flanked by inverted open reading frame (ORF) repeats, named terminal repeat long (TRL), internal repeat long (IRL), internal repeat short (IRS), and terminal repeat short (TRS) regions. The latency regions consisting of 19 ORFs (denoted UL133-151) are highlighted. They are present in the low-passage HCMV clinical strains, but are missing in the HCMV laboratory strain AD169. The HCMV-DB clinical strain is reported to share 99% genomic similarity to Toledo and JP clinical strains, but is mutated in *RL13* and *UL9* genes [11]. A previous functional annotation of these protein products showed that UL9 encodes for a membrane glycoprotein that is involved in viral growth suppression, whereas RL13 encodes for a 60S ribosomal protein. RL13a has a role in epithelial tropism.

The oncogenic phenotype of HMECs infected with the DB strain, is similar to the tumor promoting phenotype of cells infected by another clinical strain used by Fred Rapp´s group in the 1970s [29]. In addition, several HCMV gene products have shown transforming properties in single studies [30,31]. In the study of Kumar et al., the transforming properties of the HCMV DB-strain clinical isolate were compared with the AD169 laboratory strain, which has been extensively passaged and lost its ability to replicate in macrophages and epithelial cells, which is a common feature for the HCMV laboratory adapted strains. As expected, the AD169 strain did not show the same oncogenic properties as the DB-strain in vitro. The HCMV-DB strain shares close sequence similarities with the Toledo and JP clinical isolates (Figure 1) [24]. Kumar et al. investigated, in addition, the transforming properties in HMECs of another HCMV clinical strain, TB40/E, which shows 96% sequence similarity with the DB strain [24]. These results showed that, also, the TB40/E strain was able to enter the HMECs, overexpress hTERT mRNA, enhance telomerase activity, and induce some colony formation in soft agar gels. These changes were, however, significantly less pronounced than during the infection with the DB-strain, which created colonies of floating, rapidly growing, transformed cells [27].

The clinical HCMV isolates obtained from different cohorts of HCMV-infected, immunosuppressed patients and congenitally infected infants reveal high genetic variability within many important viral genes [32]. This may have consequences for their clinical manifestations and oncogenic properties. The genetic variability of HCMVs has also been observed between strains obtained from different tissue compartments of the same patients and depends on host specific factors [32]. In addition to the eventual strain variability, the epigenetic properties of the chosen primary cells may influence the susceptibility of infection to certain HCMV strains in in vitro cultures [33].

The work to investigate the transforming properties of other rarely-passaged HCMV strains is in progress [27]. However, based on historic and emerging new evidence, it is possible that certain HCMV strains may have a direct oncogenic and tumor promoting role in breast cancer, while most HCMV strains do not confer such properties. If so, these are concerning new facts, and it becomes important to understand what gene alterations that make certain HCMV strains oncogenic and also to define how prevalent such viral strains are in the society. However, first, the scientific community is awaiting conformational evidence that the oncogenic properties observed in experiments using HCMV-DB can be repeated by other researchers, and whether HCMV-DB can transform HMECs from multiple donors, since the donor cells may affect the virus’s oncogenic capability to flourish.

## 5. The HCMV Signature in Transformed Breast Epithelial Cells

HCMV IE1 and IE2 proteins are expressed during lytic infections in permeable cells, allowing the synthesis of viral particles and the lysis of the infected cells limiting their survival. The IE expression activates immunogenic pathways and is inhibited by a complex interaction between cellular transcription factors and the highly chromatinized MIEP, which is critical for the control of lytic HCMV infection [34]. As a part of the viral life-cycle, IE2 protein expression leads to an autoregulatory activation of histone methyltransferases, suppressing the MIEP [34]. During HCMV infection, a trans-localization of DMNMT1 and DNMT3a from the nucleus to the cytoplasm occurs resulting in global hypomethylation of the DNA [33]. Evidence demonstrates that TNBCs, which have high HCMV activity, exhibit extensive hypomethylation in contrast to luminal, estrogen receptor-positive cancers with lower HCMV activity [35]. This is also consistent with the results obtained from studies with the HCMV-DB clinical strain, connecting hypomethylation to HCMV activity. These studies show that the expression of numerous histone methyltransferase genes, and the genes coding for SET domain proteins with histone methyltransferase activity are downregulated in HCMV-DB infected HMECs. The same HMECs display a triple negative phenotype in comparison to uninfected HMECs [11]. An intact MIEP sequence is required for expression of IE1 and 2, efficient production of the infectious virus, and cell lysis [11]. The methylation status of the infected cells may impact on the susceptibility to productive HCMV infection [33]. On the other hand, deletion of the MIEP core promoter region from a plasmid containing the MIE genomic locus did not completely counteract IE1 and IE2 expression. Instead, it resulted in increased expression of alternative MIE transcripts, suggesting that MIEP may suppress the activity of alternative uncharacterized MIE promoters [36]. Interestingly, the MIEP sequence was reported to disappear during transformation of the HCMV-DB infected HMECs, while another part of the HCMV genome containing the HCMV lnc RNA4.9 sequence was found in the transformed cells and in the tumors [2,3,28]. Moreover, abnormal cellular localization of IE proteins in the cytoplasm and unorthodox sizes of IE proteins are often observed in cancer cells [4,22,28], indicating that the IE proteins present in tumor cells may differ from the well-studied IE1 and IE2 proteins. This could in theory be caused by inactivation and/or deletion of the MIEP in HCMV infected tumor cells.

The HCMV-induced transformation process is far from understood, but the above listed findings resemble some common features of known onco-viruses, such as human papilloma virus (HPV) and Epstein Barr virus (EBV), whose regulatory parts of the viral genome are lost during the transformation process, while some oncogenic RNA and protein products are still detected in the cancerous cells [37,38]. Mirroring the cellular transformation induced by EBV latent transcripts, expression of the HCMV latent gene transcript IncRNA4.9 has been detected in the transformed tumor cells [37].

## 6. Relevance of HCMV in the BC Microenvironment

Several studies link the increased viral activity of HCMV to a more aggressive breast cancer phenotype and a worse prognosis both in animal models and in humans [4,28,39]. According to the current view, the tumor micro-environment plays a crucial role in the development of the neoplastic process, its progression, and for the induction of the therapy resistance. HCMV displays wide tissue tropism infecting multiple cell types in the same tissues. A productive HCMV infection in cells surrounding cancer cells may, thereby, participate in the re-modelling of the tumor microenvironment. Supportive evidence for this hypothesis comes from a very recent study in a murine glioblastoma tumor model by Krenzlin et al. [40]. This study found evidence of murine cytomegalovirus (MCMV) reactivation in intra-tumoral perivascular pericytes and infected tumor cells in latently infected mice with glioblastoma. A significant reduction in overall survival was observed in infected animals compared with MCMV negative controls. The antiviral drug cidofovir improved survival in these animals by inhibiting the effects of MCMV in the tumor microenvironment [40].

HCMV infection promotes a cellular secretome that influences its micro-environment by enhancing production of important immunosuppressive mediators [41] of much relevance in cancer biology. These include the membrane-bound T regulatory (Treg) cell-expressed transforming growth factor β (TGF-β) and IL-10, which directly inhibit NK cell and T cell effector functions [42,43]. HCMV further boosts this activity by exhibiting strong immunosuppressive effects via production of an IL-10 homologue—cmvIL-10 (encoded by *UL111A* gene). cmvIL-10 can promote maturation of pro-tumoral M2 macrophages, upregulation of the proto-oncogene Bcl-3 [27], and counteract the proper maturation of dendritic cells [42]. In addition, cmvIL-10 can directly promote growth and chemotaxis of breast cancer cells [44]. Further immunomodulatory genes encoded by the HCMV secretome include UL37/vMIA; pUL144, a tumor necrosis factor (TNF) receptor homolog; and pUL128, a CC-like chemokine that modulates monocyte activity [45].

The HCMV induced secretome is, interestingly, very similar to the reported exosomal BC secretome [41,46]. The cancer cells promote M2 tissue macrophages to display an anti-inflammatory phenotype characterized by secretion of IL-6, arginase, and iNOS, and increase STAT3 signaling. This in turn promotes a local immunosuppression on tumor cells, by induced T cell anergy or through suppressive nitric oxide signaling [42]. As a matter of fact, in addition to the transforming capabilities of the HCMV-DB strain on epithelial cells, it is characterized as a highly macrophage-tropic virus. In glioblastomas, viral infection was proposed to induce an M2 phenotype that exhibited immunosuppressive effects, possibly via cmvIL-10 [42]. Another study suggests that HCMV infection of human monocytes stimulates reprogramming through engagement of the NF-κB and P13K signaling pathway, resulting in establishment of an inflammatory M1/M2 macrophage phenotype that carries signatures of both macrophage phenotypes [47]. It is somewhat unclear whether this pathway becomes active as a result of paracrine signals from HCMV infected cancer cells, such as secretion of GM-CSF and cmvIL-10, or by an active HCMV infection itself in macrophages [42]. The M2 macrophages display an immunosuppressive phenotype that is closely related to tumor-associated macrophages (TAMs), as shown by upregulation of p-STAT3, downregulation of p-STAT1, and secretion of TGF-β and IL-10 [42,47]. CMV infection induced STAT3, and induces production of TGF-β, IL-10, and cmvIL-10 [42]. Thus, infections with HCMV may contribute to neoplastic transformation of the breast epithelial cells, and also to recruitment and differentiation of M2-macrophages into the same tissue compartment.

In vitro studies performed with several cancer cell lines indicate that HCMV infection in the tumor microenvironment can induce stemness and an epithelial–mesenchymal transformation (EMT) program, thereby, potentially increasing the aggressiveness of tumors [48]. A recent publication reported that latent CMV infection in a mouse xenograft model resulted in increased numbers and sizes of lung metastases and enhanced cancer cell proliferation in the metastatic nodules [39]. Furthermore, Oberstein and Shenk recently showed that HCMV infection of two breast cancer cell lines in vitro inhibited EMT while promoting mesenchymal–epithelial transition (MET), indicating that circulating HCMV infected cancer cells can undergo the reversed transition MET necessary to establish distant metastases [49]. This hypothesis is further supported by data suggesting that over 96% of brain metastases derived from breast cancer are HCMV protein positive, while surrounding tissues are virus negative [4]. In addition, the gene expression of alpha 6 integrin (ITGA6), a marker of stemness and the proto-oncogene tyrosine-protein kinase Src, are upregulated in HCMV-DB-infected HMECs compared to uninfected HMECs [11]. In BC samples, increased expression of GM-CSF is associated with an increase in CCL18-secreting macrophages and the realization of EMT by cancer cells [50]. We have shown previously that high-grade positivity for HCMV-IE (>50% of the cancer cells) correlated negatively with ER and PgR expression in human breast tumors [28]. Several studies confirm the correlation of a high prevalence of immunosuppressive macrophages with HR-negativity and high mitotic rate in BC [2,3,4,23]. Thus, the mutual interaction between the tumor initiating cells and M2 macrophages in BC could increase tumor invasiveness and metastatic potential, and is clinically associated with lower patient survival rates [51]. HCMV infected cells secrete MCP-1/CCL2 [52], which is a potent factor that attracts monocyte/macrophages into tissues. HCMV protein expression is evident in tumor-associated macrophages in tumors, but their functional role in disease progression needs further investigation.

## 7. HCMV, M2, and T Regulatory Cells

**Figure 2:**
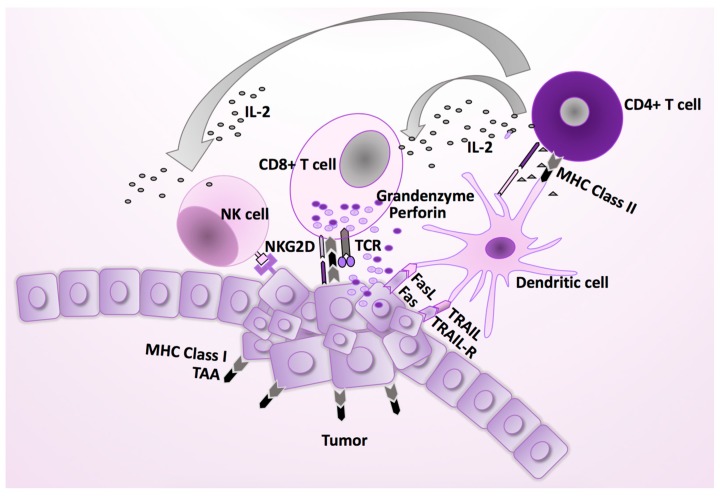
The normal immune response against virus infections. Cytotoxic CD8+ T cells (CTLs) are effector cells, which display a critical role in the immune defense against intracellular pathogens, including HCMV, and in tumor surveillance. They can recognize viral or mutant cellular peptides presented by MHC class I molecules on their target cells, and exclusively eliminate the virally infected or malignant cells. Their function is supported by antigen specific CD4+ T cells, which are activated by an antigenic peptide presented by MHC class II histocompatibility complexes on the antigen-presenting cells (APCs), such as macrophages and dendritic cells. In addition to inducing a B cell dependent antibody response, CD4+ T cells are crucial for the initial antigenic activation of naïve CD8+ T cells, which contribute to the pool of sustaining memory CD8+ T cells. IL-2 production from antigen stimulated CD4+ T cells activates natural killer (NK) cells, which play an important role in the immune surveillance against HCMV infected or malignant cells, including HCMV infected macrophages [18,19]. FasL, Fas ligand; TRAIL-R, tumor necrosis factor (TNF)-related apoptosis-inducing ligand; TCR, T cell receptor; NK, natural killer cell; MHC, major histocompatibility complex.

**Figure 3:**
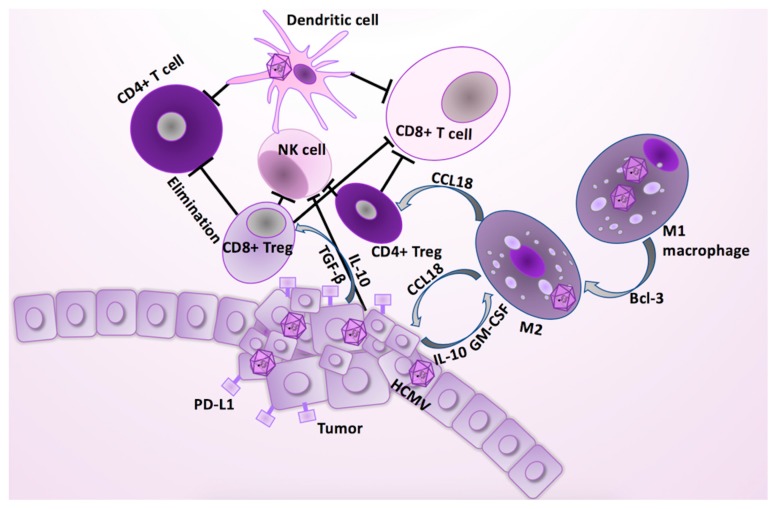
HCMV infection affects several cell types in the tumor. HCMV infection in epithelial cells is pro-tumorigenic, and contributes to the polarization of M1 to M2 macrophages or TAMs. The key TAM-mediated immunosuppressive effects are related to TAMs’ ability to recruit the CD4+ T regulatory (Treg) cells. In addition, tumor cells secrete immunosuppressive cytokines, such as TGF-β and IL-10, which are conductive to proliferation of the TAMs and induction of the CD8+ Treg cells [43]. TAM/Treg shift results to unfavorable, immune suppressive effects, resulting in tumorigenic tolerization and immune escape, thereby promoting cancer progression [43,53]. The CD8+ Tregs can eliminate the activated tumor specific CD4+ T cells, and inhibit actions of both CD8+ T cells and NK cells. The CD4+ Tregs can counteract both the tumor specific CD8+ CTL, and B cell activation and proliferation. HCMV infection and IL-10 secretion from the tumor cells promote an immature phenotype in dendritic cells, which contributes to the reduced antigen-presenting capacity, and increased elimination of the activated T cells through Fas/FasL and TRAIL pathways [42]. The tumor escape is supported when the aberrant activation of the T-cell receptor alone in mature T cells produces a long-lived state of functional unresponsiveness, anergy. Tumor cells express death ligands (PD-L1), of which receptors are expressed on the surface of the immune cells, contributing to the lower proliferation, cytokine production, and their cytotoxic abilities.

A key function of TAMs is to mediate immune suppressive effects by recruiting Treg cells (Figure 2 and Figure 3). Treg cells consist of diverse subsets of immunosuppressive cells, both arising from CD4+ and CD8+ T cells, which play an essential role in regulating antigen specific cytotoxic immune responses and preventing autoimmunity. Once present in the tumor micro-environment, the TAM/Treg shift leads to unfavorable immunosuppressive effects. By recognizing the same antigens as effector T cells, Treg cells are associated with poor immune responses to tumor antigens, and thereby they contribute to induction of tumorigenic tolerance and immune escape that may further promote cancer progression [53]. Both the HCMV-infected cells and the cancer cells secrete TGF-β, which may promote breast tumor metastasis in vivo through several mechanisms [43]. TGF-β1 levels correlate positively with the percentage of CD8+ Treg cells in ovarian cancer, and high TGF-β1 expression triggers the suppressive function of in vitro-induced CD8+ Treg cells. It was recently reported that TGF-β1 secreted by ovarian cancer cells could generate CD8+ Treg cells in vitro from CD8+ T cells through engagement of the p38 MAPK signaling pathway [54]. TGF-β1 secreted by the tumor cells and CMV-infected macrophages might be an imperative factor for the differentiation of CD8+ Treg cells from effector CD8+T cells, thereby shifting the immune balance within the tumor from cytotoxic to an immunosuppressive state.

Clinically, an increased frequency of Tregs is associated with BC progression, histological grade, estrogen receptor negativity, HER-2 positivity, and molecular subtype in BC [55]. HER-2 positive BC and TNBC subtypes have significantly higher amounts of Treg cells, and decreased levels of CD8+T cells compared with the luminal subtype [55]. The extent of Treg cells also predicts worse outcome in TNBC patients [56]. Complete clinical response is associated with the disappearance of Tregs in breast carcinoma, further supporting evidence of substantial immuno-suppressive capabilities of Tregs in the breast tumor microenvironment [57]. The extent of the presence of CTLs similarly predicts improved responses to certain therapies, including chemotherapy, particularly in TNBC, and to trastuzumab in HER-2 positive BC. This also applies to the pathological complete response rate to chemotherapy during the neoadjuvant treatment and overall response and survival of metastatic disease [58].

Treg cells may control the latent CMV infection in tissue specific and cell type specific manners as shown recently in the murine model of CMV (MCMV) infection [59]. In the spleen, Treg cells antagonized CD8+ T cell effector functions and promoted viral persistence and replication, while in the salivary glands, Tregs prevented IL-10 production and limited viral reactivation and replication. Removal of Treg cells resulted in enhanced elimination of the virus by CTLs in the mouse spleen [59]. Unexpectedly, in the same animals, ablation of Tregs resulted in a significant increase in virus production in the salivary gland, which was accompanied by augmented local IL-10 production by Foxp3-CD4+T cells [59]. Furthermore, neutralization of IL-10 after Treg depletion significantly decreased viral load in the salivary gland [59]. These data indicate that HCMV protects its own existence during latency by recruitment of Tregs to suppress the CD8+ CTLs, to avoid virus elimination. Thus, the immune-evasion mechanisms observed in breast cancer by abrogated CD8+ T cell effects, and recruitment of Tregs resembles that observed during latent MCMV infection. Since the regulatory T cell response to HCMV may be tissue specific as in MCMV infection, manipulation of the general HCMV specific Treg functions may give contrasting consequences for HCMV reactivation in different tissues in this context [59].

## 8. Treatment of BC by Targeting Immunosuppressive Cells in the Tumor Micro Environment

Several potential therapeutic strategies targeting the TAMs are currently under investigation, but have not been yet introduced in clinical practice. Importantly, such strategies could also revitalize T cells under control of HCMV, thereby augmenting their ability to kill HCMV infected tumor cells. While there is much interest in research into individualized medicine in breast and other cancers, there appear to be very few ongoing clinical studies investigating the use of anti-HCMV approach in BC therapy. We propose that this would be a rational thing to do, since previous antiviral treatment targeting HCMV in patients with glioblastoma (GBM) and the use of DC vaccinations targeting HCMV have given promising results [60,61], as has adaptive, anti-HCMV targeted T cell therapy in recurrent GBM patients [62]. Similarly, T effector cells stimulated with autologous DCs pulsed with CMV pp65 RNA have shown anti-tumoral effects associated with extended survival rates of GBM patients [63].

## 9. Conclusions

In breast cancer, especially TNBC, the tumor cells frequently express several HCMV gene products, which may be a result of an oncogenic infection in breast epithelial cells. Such infections may be caused by certain CMV strains with the oncogenic capacity to transform tumor cells. However, HCMV also affects several other cell types in the tumor; it is pro-tumorigenic; and it contributes to the polarization of M1 to immunosuppressive M2 macrophages. The immunosuppressive macrophages and tumor cells induce tumorigenic tolerance by suppressing tumor-specific immune reactions by inducing regulatory T cells. This occurs through the inactivation and deletion of immune cells, and by inducing tolerization by cytokines or cross-presentation related to dendritic cells and macrophages. The impact of the individual tumorigenic and immunogenic components in BC may vary, contributing to the wide diversity of disease characteristics along with the different activity levels of HCMV in the tumor cells. As the key disease mechanisms of BC include the same abnormal immunity that can be induced and regulated by HCMV to promote its own survival, anti-HCMV treatment in BC may potentially target a wide range of the disease components. It is therefore rational to seek opportunities to evaluate anti-viral therapy targeting the virus-mediated effects in tumor cells; e.g., in addition to established therapies, to assess whether this treatment proves efficient at improving the outcomes of breast cancer patients, particularly in TNBC patients whose treatment options today are still very limited.
